# Long-term whole-body vibration induces degeneration of intervertebral disc and facet joint in a bipedal mouse model

**DOI:** 10.3389/fbioe.2023.1069568

**Published:** 2023-03-17

**Authors:** Lin-Yu Jin, Hong-Lin Yin, Yi-Qiong Xu, Shuai Xu, Xiao-Xing Song, Yan Luo, Xin-Feng Li

**Affiliations:** ^1^ Department of Orthopedics, Shanghai Key Laboratory for Prevention and Treatment of Bone and Joint Diseases, Shanghai Institute of Traumatology and Orthopedics, Ruijin Hospital, Shanghai Jiao Tong University School of Medicine, Shanghai, China; ^2^ Department of Spine Surgery, Peking University People’s Hospital, Beijing, China; ^3^ School of Materials Science and Engineering, Shanghai Jiaotong University, Shanghai, China; ^4^ Department of Anesthesiology, Ruijin Hospital, Shanghai Jiaotong University School of Medicine, Shanghai, China

**Keywords:** biomechanics, intervertebral disc (IVD) degeneration, facet joint degeneration, whole body vibration, anabolism and catabolism

## Abstract

**Background:** Whole body vibration (WBV) has been used to treat various musculoskeletal diseases in recent years. However, there is limited knowledge about its effects on the lumbar segments in upright posture mice. This study was performed to investigate the effects of axial Whole body vibration on the intervertebral disc (IVD) and facet joint (FJ) in a novel bipedal mouse model.

**Methods:** Six-week-old male mice were divided into control, bipedal, and bipedal + vibration groups. Taking advantage of the hydrophobia of mice, mice in the bipedal and bipedal + vibration groups were placed in a limited water container and were thus built standing posture for a long time. The standing posture was conducted twice a day for a total of 6 hours per day, 7 days per week. Whole body vibration was conducted during the first stage of bipedal building for 30 min per day (45 Hz with peak acceleration at 0.3 g). The mice of the control group were placed in a water-free container. At the 10th-week after experimentation, intervertebral disc and facet joint were examined by micro-computed tomography (micro-CT), histologic staining, and immunohistochemistry (IHC), and gene expression was quantified using real-time polymerase chain reaction. Further, a finite element (FE) model was built based on the micro-CT, and dynamic Whole body vibration was loaded on the spine model at 10, 20, and 45 Hz.

**Results:** Following 10 weeks of model building, intervertebral disc showed histological markers of degeneration, such as disorders of annulus fibrosus and increased cell death. Catabolism genes’ expression, such as Mmp13, and Adamts 4/5, were enhanced in the bipedal groups, and Whole body vibration promoted these catabolism genes’ expression. Examination of the facet joint after 10 weeks of bipedal with/without Whole body vibration loading revealed rough surface and hypertrophic changes at the facet joint cartilage resembling osteoarthritis. Moreover, immunohistochemistry results demonstrated that the protein level of hypertrophic markers (Mmp13 and Collagen X) were increased by long-durationstanding posture, and Whole body vibration also accelerated the degenerative changes of facet joint induced by bipedal postures. No changes in the anabolism of intervertebral disc and facet joint were observed in the present study. Furthermore, finite element analysis revealed that a larger frequency of Whole body vibration loading conditions induced higher Von Mises stresses on intervertebral disc, contact force, and displacement on facet joint.

**Conclusion:** The present study revealed significant damage effects of Whole body vibration on intervertebral disc and facet joint in a bipedal mouse model. These findings suggested the need for further studies of the effects of Whole body vibration on lumbar segments of humans.

## Introduction

As the aging population increases, low back pain (LBP) is becoming one of the central issues in global healthcare, and LBP also imposes a heavy financial and psychological burden on society and patients (GBD, 2017 Disease and Injury Incidence and Prevalence Collaborators et al., 2017). LBP is not a simple event induced by a single specific factor, but a pathological combination involving vertebrae, intervertebral discs (IVD), facet joints (FJ), muscles, and ligaments (Ekşi et al., 2020). Furthermore, epidemiologic research has found that IVD degeneration (IVDD) and FJ degeneration (FJD) commonly existed in degenerated spine diseases and furthermore are the major reasons for LBP (Seidler et al., 2009; Li et al., 2011a). As an exquisite 3-dimension architecture, IVD is composed of annulus fibrosus (AF) made from fibrocartilage, nucleus pulposus (NP) containing collagen II and proteoglycan, and end plate (EP), an important nutritional channel composed of hyaline cartilage (Jin et al., 2018). As well, FJ is the only synovial joint of the spine, and plays as an articulated structure between lamina behind vertebrae (Li et al., 2011a). IVD and FJ together constitute the three-joint complex of the spine, which serves as a shock absorber system for the spine and transfers axial loads, and weakens energy imposed on the structures of the spine (Dowdell et al., 2017). The pathological process of IVDD and FJD generally initiate from progressive loss of proteoglycans, water, and nutrition, directly resulting in irreversible mechanical changes in NP, fissures/bulging in AF, decreased height of IVD, and narrowed space and cartilage degeneration of FJ (Borenstein, 2004; Manchikanti et al., 2008; Kague et al., 2021). Therefore, investigating the pathophysiological mechanism of IVDD and FJD is of great significance.

Mechanical loading is an important factor to maintain the homeostasis of IVD and FJ, and to retain the healthy extracellular matrix (ECM) of IVD and FJ (Lee et al., 2003; Wong and Carter, 2003; Chen et al., 2004). Previous studies have shown that physiologic mechanical loading could lead to a higher level of ECM anabolism, whereas over-mechanical-loading conditions promoted the level of ECM catabolism and furthermore IVD and cartilage degeneration (MacLean et al., 2003; Wang et al., 2007; Wuertz et al., 2009). Region-specific responses of NP and AF to mechanical stimulations have been studied with different responses to frequency and magnitude (Maclean et al., 2004). It is well known that physiologic mechanical loading is essential for IVD and FJ health (Belavý et al., 2011; Jaumard et al., 2011). However, specific parameters of mechanical loading from occupational exposure to IVD and FJ health have not been established. Exposure to the whole-body vibration (WBV) of daily life has been established as a factor in the development of spinal pathologies, such as disc herniation and nerve damage in human beings (Patterson et al., 2021), and spinal segmental injury from WBV is most likely to happen at the resonant frequency. Furthermore, the effects of WBV on IVD and FJ degeneration might be species-dependent, as resonant frequency is a function of animal/tissue mass (Patterson et al., 2022). Within the last decades, high-frequency (20–90 Hz) WBV has been used as a clinical therapy for various diseases including bone fracture, osteoporosis, spinal cord injury, and multiple sclerosis (Rubin et al., 2004; Herrero et al., 2011; Claerbout et al., 2012; Pang et al., 2013). WBV loading condition was reported to be associated with molecular and structural changes in IVD. For example, short-time high-frequency WBV (45 Hz) application promoted the expression of anabolic genes and suppressed the expression of catabolic genes in a frequency-dependent manner (McCann et al., 2013). While, in a long-term WBV application (30 min per day, 45 Hz, 5 days per week, 4 weeks in total), they demonstrated negative effects of WBV on IVD and knee joint tissues (McCann et al., 2015). The dichotomy results demonstrate that the adverse effects of high-frequency WBV on musculoskeletal structures have not been carefully studied until now. In addition, to our best knowledge, the effects of WBV on the facet joint have not been studied well until now.

The mouse has become a widely-used animal model to explore the underlying mechanism of IVDD and FJ degeneration. However, the orientation of the quadruped spine is quite different from human beings during daily life. Bipedal standing posture (axial mechanical loading) is an important mechanical loading condition for IVD and FJ, where IVDs carry the majority of the axial load and FJs bear the remaining load. Both IVD and FJ depend on each other’s health to transmit the load placed on the lumbar spine. As the disc height decreases due to IVDD, the load-bearing role of FJs increases (Dunlop et al., 1984; Schmidt et al., 2007; Schmidt et al., 2008). Similarly, the IVD benefits from the effects of FJ on limiting motion and thereby decreasing the strain on NP and AF (Ao et al., 2019; Li et al., 2020). Thus, it is important to use a bipedal animal model to mimic the standing posture of humans as well as investigate the effects of high-frequency WBV conditions on IVD and FJ. Previous studies demonstrated a novel bipedal mouse model by placing mice in limited water to induce a standing posture for longperiods, and thus directly resulting in IVDD and FJ degeneration (Ao et al., 2019; Li et al., 2020). Here, the aim of the present study is to explore the effects of long-term WBV on the catabolic and anabolic metabolism of IVD and FJ using the novel bipedal mouse model. Our hypothesis is that high-frequency WBV could generate harmful effects on the anabolic and catabolic balance of IVD and FJ in a bipedal standing posture.

## Methods and materials

### Mouse model and study design

**FIGURE 1:**
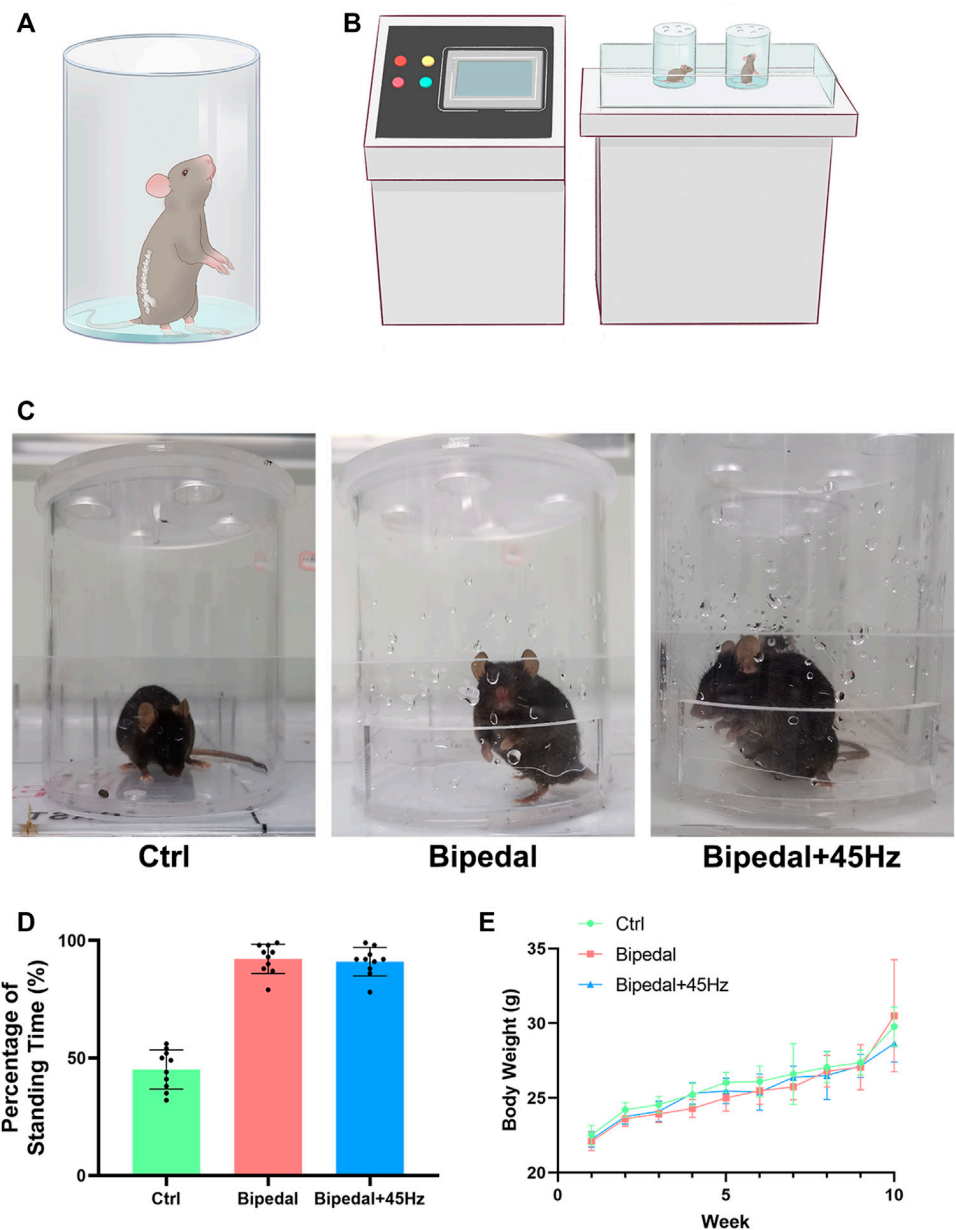
**(A)** A diagram of bipedal mice that were placed in the water-containing space to keep the standing position. **(B)** Diagram of WBV loading conditions on the bipedal mice. **(C)** Example image of control mice, bipedal mice, and bipedal+45 Hz mice. **(D)** The percentage of time in the standing posture of the three groups during the treatment sessions was then calculated and compared. **(E)** The body weight of the three groups during the treatment.

Thirty C57BL/6 male mice (6 weeks old) were purchased from Shanghai JieSiJie Laboratory Animal Co., Ltd. (Shanghai, China). All mice were housed in a standard animal room with constant temperature and humidity. The mice were randomly divided into three groups: control (Ctrl), Bipedal, and Bipedal+45 Hz (*n* = 10 mice for each group). We took advantage of the water-escape instinct of mice (shown in Figure 1A) to induce the bipedal standing posture according to the work of Ao (Ao et al., 2019). In brief, the mice of bipedal/bipedal+45 Hz groups were placed in a rubber chamber containing a small amount of water to induce the bipedal posture for relatively long-term standing (Figure 1C), and the mice of the control group were treated similarly to the other two groups except that no water was placed in the chamber. The chamber has sufficient space, ventilation, and light, with or without the presence of room temperature water to push the mouse to stand. The mice of these three groups underwent interventions with/without water in the chamber 7 days a week and two rounds of 3 h each day (a total of 6 h). Mice revieved 2 h of free activity for between the two daily standing-posture-building sessions in normal feeding cages with free intake of food and water. For the mice in the Bipedal+45 Hz group, the parameters of WBV mainly referred to previous studies (McCann et al., 2013; McCann et al., 2015). Briefly, the chambers containing the mice of the Bipedal+45 Hz group were placed on a vibration platform with vertical sinusoidal vibration at 45 Hz (amplitude of 74 μm, peak acceleration at 0.3 g) for 30 min per day at the beginning of Bipedal model building (Figure 1B). The body weight of these three groups were recorded weekly. All mice were euthanized using carbon dioxide after ten weeks of experimentation to assess the degree of IVDD and FJD.

### Micro-CT scanning and disc height index (DHI) analysis

One important indicator of IVDD is the decreased height of the intervertebral disc. After the mice were euthanized, lumbar spine segments (L3-L6) were collected from these groups and fixed with 4% paraformaldehyde for 24 h. Then, the fixed spines were scanned using micro-CT (SkyScan 1072; Bruker micro-CT). The DHI of three segments (L3/L4, L4/L5, L5/L6, in total 15 discs for each group) was calculated by averaging the measurements of the anterior, middle, and posterior portions of the IVD and dividing that by the average of adjacent vertebral body heights. Two investigators were blinded to the treatment groups to calculate the DHI.

### Histological analysis

The fixed lumbar spinal segments (L4-L5, *n* = 5 for each group) and fixed lumbar FJ segments (*n* = 4 for each group) were decalcified for 21 days in 10% EDTA, and then were embedded using paraffin and sectioned using a 4 μm micro-tome (Leica microsystem). Then several spinal segments from each group were sagittally sectioned for IVD staining. The remaining spinal segments of each group were transversely sectioned for the FJ staining. According to the manufacturer’s protocol, these slides were stained with hematoxylin and eosin (HE), Safranin O-fast Green, and/or picrosirius red. Sections were imaged using a Leica microscope system that was equipped with a polarizing filter. HE staining slides were evaluated according to the modified Thompson grading system (Thompson et al., 1990). In brief, the system contained a 5-category grading scale to assess the nucleus, annulus, and endplate. Each slide was scored independently according to the morphologic features of HE staining.

### Immunohistochemistry (IHC)

For IHC analysis, the slides were baked for 50 min at 60 °C, then rehydrated in xylene and graded ethanol, and finally washed with water, with 5 min in each step. The endogenous peroxidase activity was eliminated by using 3% H_2_O_2_, and phosphate-buffered saline was used for washing these slides. After retrieval and blocking, these slides were incubated with antibodies of anti-Collagen II (1:200, Abcam, United States), anti-Collagen I (1:200, Abcam, United States), anti-Collagen X (1:250, Affinity, United States), anti-Aggrecan (1:200, Abcam, United States), anti-MMP13 (1:250, Affinity, United States), anti-Adamts4 (1:250, affinity, United States), and anti-Adamts5 (1:250, affinity, United States) at 4°C overnight respectively. After washing with PBS triple times, these slides were incubated with peroxidase-conjugated secondary antibody for 2 h at room temperature. Finally, these slides were incubated in ABC complex for 30 min. Staining was detected with DAB peroxides substrate solution for 3 min, followed by rinsing in distilled water. The sections were dehydrated by graded ethanol, cleared in xylene, and mounted with permount medium after counterstaining with Gill’s hematoxylin solution for 3 min. The results were captured by a digital microscope (Leica, German). The semiquantitative analysis of IHC (HSCORE method) was measured according to previous studies (Budwit-Novotny et al., 1986; Li et al., 2011b; Song et al., 2021). Briefly, for each sample, three tissue sections from each sample were randomly obtained, and under the microscope (Leica, German) with ×200original magnification, five randomly selected areas were evaluated for each tissue slide. The percentage of the cells at each intensity group within these areas was determined by two investigators blinded to the type of tissues. The average score was used.

### Terminal deoxynucleotidyltransferase (TdT)-mediated deoxy UTP nick-end labeling (TUNEL) assay (cell death analysis)

IVD were assessed for *in situ* cell death using a TUNEL staining kit according to the manufacturer’s protocol (Roche, United States). DAPI was used to label the nucleus and imaged with a fluorescence microscope (Leica, German). The TUNEL-positive cells were counted for the NP and AF of IVD by two independent blinded observers. The results were expressed as a percentage of the total cells.

### RNA extraction and real-time quantitative polymerase chain reaction (qPCR)

At the end of 10 weeks of these models, thoracic IVDs (T10-L3) from each mouse (n = 6 of each group) were isolated by microdissection. The IVDs were ground thoroughly in liquid nitrogen and immediately placed into TRIzol reagent (Thermofisher, Life Technology). Then, RNA was extracted according to the manufacturer’s protocol, quantified using NanoDrop (Thermofisher, United States), and 0.5 μg was reverse transcribed into cDNA using a reverse kit (TAKARA, Japan). Gene expression was evaluated by qPCR using a Roche LightCycler^®^ 480II (Roche, Diagnostics). PCR amplification was performed with TB green premix Ex Taq (Takara) according to the manufacturer’s instructions. The forward and reverse primer sequences were shown in Table 1. The progress of qPCR contained two steps: first, an initial 30s of heating to 95°C, then 20 cycles consisting of 5s at 95°C followed by 20 s at 60°C. The ΔΔCt method was used to evaluate the transcript levels, and data were normalized for input based on β-actin and expressed relative to mice in the control group.

**TABLE 1:** Primer sequences for real-time PCR.

Gene	Forward primer, 5′-3′	Reverse primer, 5′-3′
Acan (Aggrecan)	CCT​GCT​ACT​TCA​TCG​ACC​CC	AGA​TGC​TGT​TGA​CTC​GAA​CCT
Col 1a	GCT​CCT​CTT​AGG​GGC​CAC​T	CCA​CGT​CTC​ACC​ATT​GGG​G
Col 2a	CAG​GAT​GCC​CGA​AAA​TTA​GGG	ACC​ACG​ATC​ACC​TCT​GGG​T
Mmp13	CTT​CTT​CTT​GTT​GAG​CTG​GAC​TC	CTG​TGG​AGG​TCA​CTG​TAG​ACT
Adamts4	ATG​GCC​TCA​ATC​CAT​CCC​AG	AAG​CAG​GGT​TGG​AAT​CTT​TGC
Adamts5	GGA​GCG​AGG​CCA​TTT​ACA​AC	CGT​AGA​CAA​GGT​AGC​CCA​CTT​T
β-actin	GGC​TGT​ATT​CCC​CTC​CAT​CG	CCA​GTT​GGT​AAC​AAT​GCC​ATG​T

### Finite element model building and whole spine vibration test

The images of mouse lumbar spine (L4-L6) obtained from micro-CT were subjected to noise elimination, and binarization was performed using thresholds obtained by discrimination analysis. The finite model of bone tissue and intervertebral disc were reconstructed from the segmentation of trabecular microstructure. For the bone tissue model, due to a large number of trabecular micro structures, the use of a geometric solid model may lead to topological errors and poor geometric characteristics. Therefore, geometric repair and optimization operations are needed for preprocessing. The finite element mesh models can be directly discretized by 10 nodes of quadratic tetrahedral elements from the geometry solid models. The ligaments were truss elements and were simulated by T3D2 elements in this model. The insertion points and areas were closely matched with published data (Jerome et al., 2018; Zheng et al., 2021). The material properties of tissues were set according to Table 2 and previous research (Lambers et al., 2013; Thiagarajan et al., 2014; Kim et al., 2015; Nyman et al., 2015; Zheng et al., 2021). Then, the analysis of the finite element model was carried out in ABAQUS software (Waltham, United States). All degrees of freedom of the inferior surface of the L6 vertebral body were fixed. To mimic spinal vibration loading conditions, a sinusoidal vertical load of 0.2N(37), at three different frequencies of 10, 20, and 45 Hz respectively, was imposed on the superior surface of L4 vertebrae according to the *in vivo* loading conditions. The transient dynamic analyses were performed on the model using ABAQUS.

**TABLE 2:** Material properties of spinal structures and instrumentations.

Description	Element type	Young’s modulus (MPa) E	Poisson ratio
Bone	Shell elements	148,000	0.3
Facet cartilage	3-D solid elements (4 nodes)	10.4	0.4
Annulus fibers	3-D solid elements (8 nodes)	35	0.2
Nucleus pulposus	3-D solid elements (8 nodes)	1.0	0.2
Endplate	3-D solid elements	100	0.2
Ligament	Truss element	15	0.3

### Statistical analysis

All the data were presented as mean ± SD, and the differences between the control group and treated groups were analyzed by one-way ANOVA methods and *post hoc* analysis was carried out with the Bonferroni test. A *p*-value <0.05 was considered statistically significant. Statistical analysis was performed using the SPSS (IBM, 22.0, United States) software.

## Results

To examine the effects of long-term exposure to WBV on the intervertebral disc and facet joint of the spine, a novel bipedal mouse model was used and exposed to vertical sinusoidal WBV for 10 weeks. The parameters of vibration were based on the current clinical and exercise guidelines (30 min for each day, 7 days for a week, and 45 Hz with peak acceleration at 0.3 g). Then, the IVDs and FJs were evaluated and compared to those of age-matched control mice and bipedal mice.

Taking advantage of the water-escape behavior of mice (Figure 1A), the bipedal model was successfully built in our study. In addition, the whole-body vibration parameters we used did not influence the habit and standing posture of mice (Figures 1B, C). To confirm whether the mice in the Bipedal+45 Hz spent similar time on their hindlimbs compared to the Bipedal group, and spend more time bipedally than the control group, the time was recorded in the process of model building. The percentage of bipedal time during the model of these three groups was then calculated and compared. Compared with the ctrl group, the Bipedal group and Bipedal+45 Hz group mice spent more time in the standing posture (*p* < 0.001), and there was no difference in the standing time between the Bipedal group and Bipedal+45 Hz group (*p* > 0.05, Figure 1D). These results indicated that the mice in the Bipedal+45 Hz group effectively maintained the standing posture compared with the ctrl group in the chambers. Furthermore, the mouse body weight of the bipedal group and Bipedal+45 Hz group did not show statistical differences compared with that of the control group mice (Figure 1E).

**FIGURE 2:**
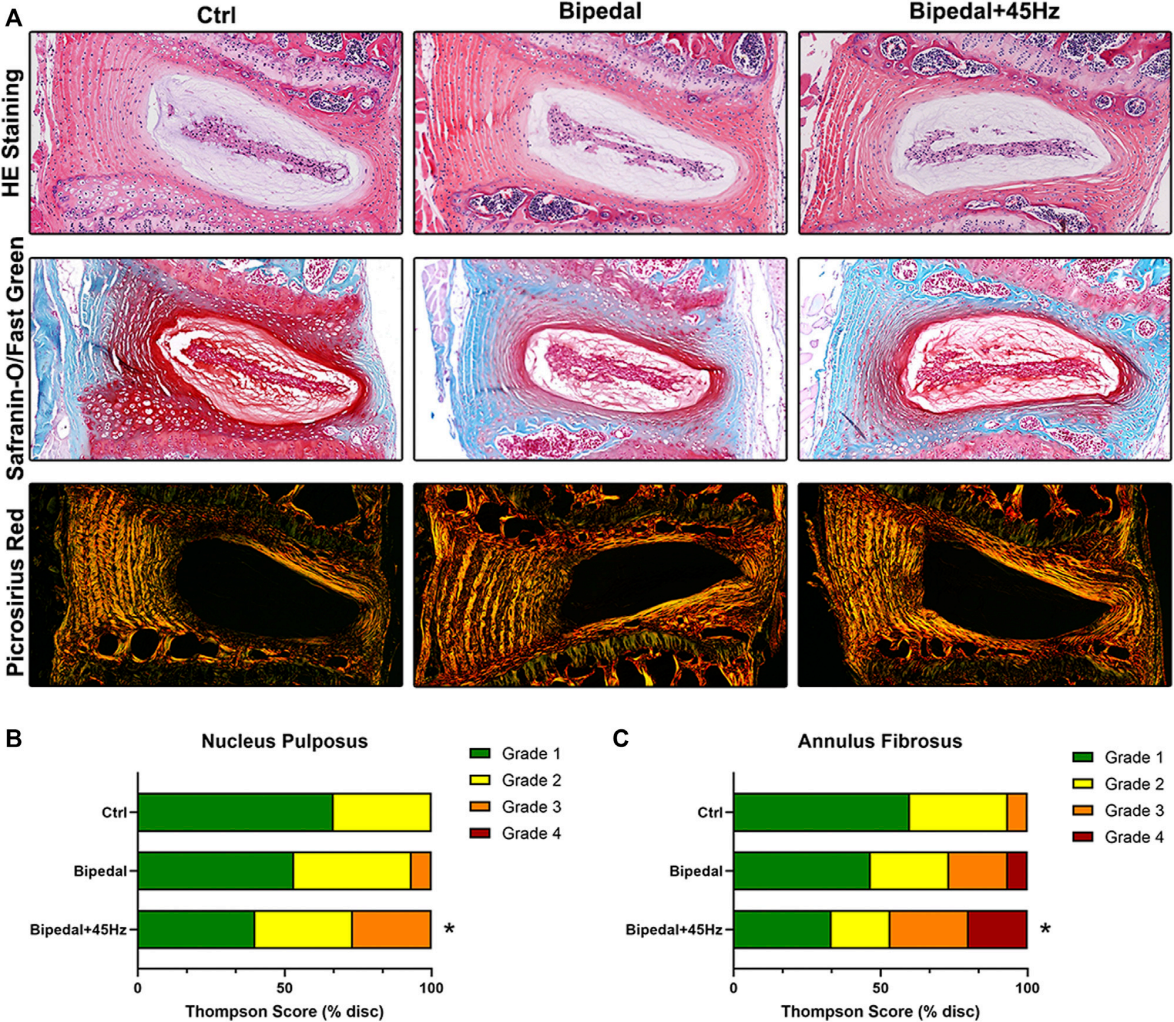
**(A)** The HE staining and safranin-O/Fast green staining results showed that the number of NP cells and the amount of extracellular matrix decreased, and the arrangement of the AF was disordered in the Bipedal and Bipedal+45 Hz groups. Picrosirius Red staining results were observed under polarized light microscopy. These results showed that disrupted collagen organization in the AF was apparent in bipedal+45 Hz mice. **(B, C)** Histological scores on the modified Thompson grading scale. Evaluation of the morphologic grade of tissue degeneration demonstrated a significant increase in tissue degeneration in the annulus fibrosus (AF) of mice subjected to WBV. Data are representative of five IVDs per mouse. *p < 0.05 compared with control mice.

Firstly, micro-CT was used to assess the height of the lumbar intervertebral disc and the results of DHI showed that after 10 weeks of procedures of the bipedal model (Supplementary Figure S1), there were statistical differences between the control group and Bipedal group (4.772 ± 0.376 vs. 4.189 ± 0.556, *p* = 0.0039) or between the control group and Bipedal+45 Hz group (4.772 ± 0.376 vs. 3.9864 ± 0.5157, *p* = 0.0001). There was a decreased tendency of DHI in the Bipedal+45 Hz group compared to the Bipedal group, but no statistical difference was found. Furthermore, histologic results of HE staining revealed that several degenerative conditions, such as decreased number of NP cells and the number of ECMs, thinner and disordered fibers of AF, were observed in the bipedal group compared to the control group. In addition, the WBV treatment aggravated these degenerative conditions of IVD (Figure 2A). Safranin O-fast green staining demonstrated the following phenomenon: (GBD, 2017 Disease and Injury Incidence and Prevalence Collaborators et al., 2017): loss of distinct boundary between NP and AF, and (Ekşi et al., 2020) decreased amount of mucinous material leading to weak glycosaminoglycan staining in the IVDs of the bipedal group compared to that of the control group. Similarly, WBV enhanced the degeneration of IVDs compared to the bipedal group. Furthermore, picrosirius red staining revealed disrupted collagen organization using polarized light microscopy. Disordered collagen lamellae and widening of the interlamellar spaces were found in the AF of the bipedal group compared to the control group, as well as, the WBV treatment also aggravated these disorders of AF in the bipedal+45 Hz group. A Thompson scoring system was used to assess the histologic appearance of IVDs among these groups. The scores indicated significant degenerative changes in the AF of bipedal mice compared to the controls, and WBV treatment enhanced the degenerative process of AF in the bipedal+45 Hz group compared to the bipedal group (Figures 2B, C).

**FIGURE 3:**
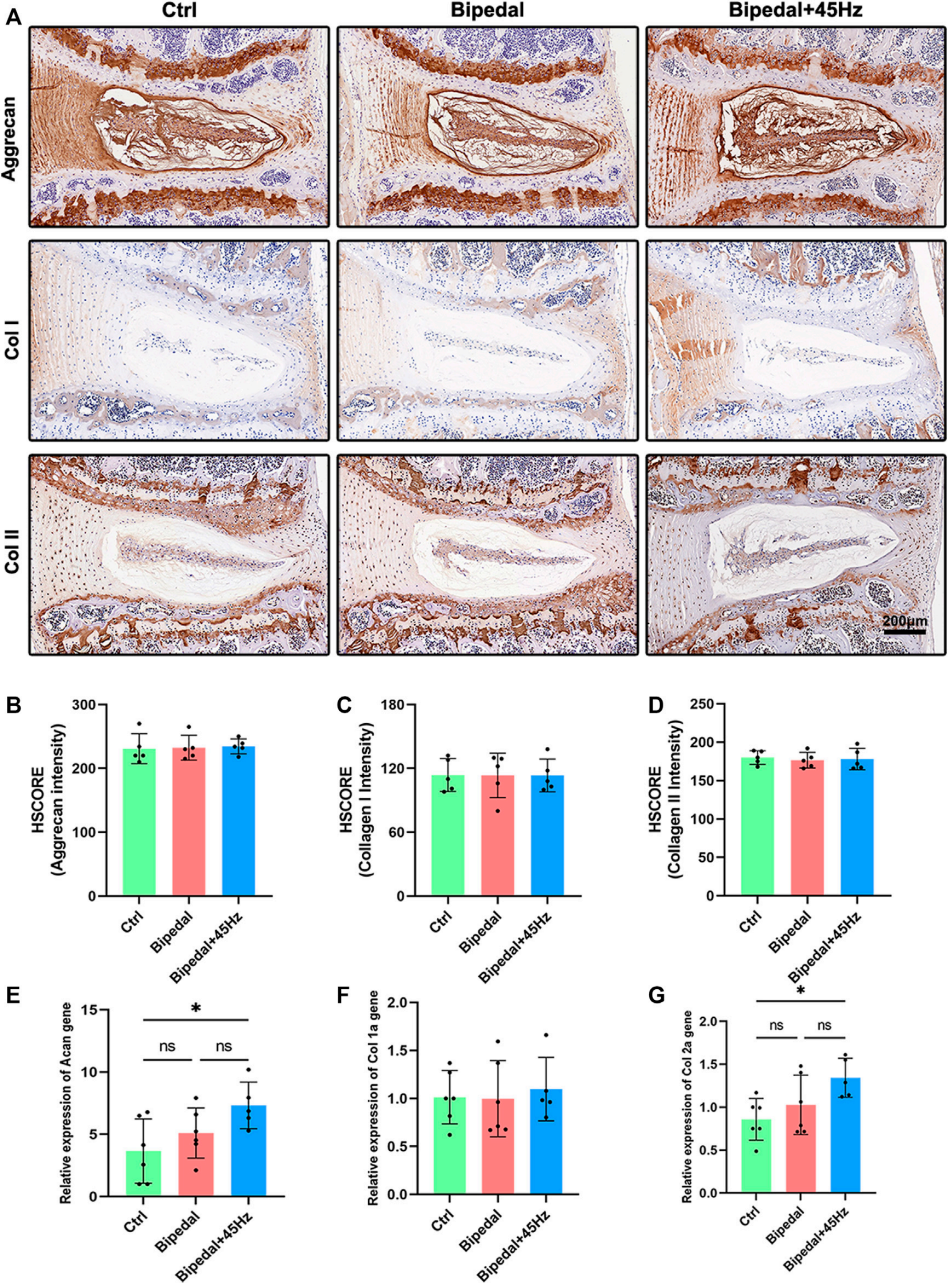
**(A)** The anabolic synthesis (aggrecan, collagen I, and collagen II) of IVD was marked using immunohistochemistry staining of these three groups. **(B–D)** The HSCORE results of these markers (*n* = 5 discs per group). **(E–G)** The analysis of IVD anabolic gene expression using qRT-PCR (*n* = 6 mice per group). *p < 0.05.

**FIGURE 4:**
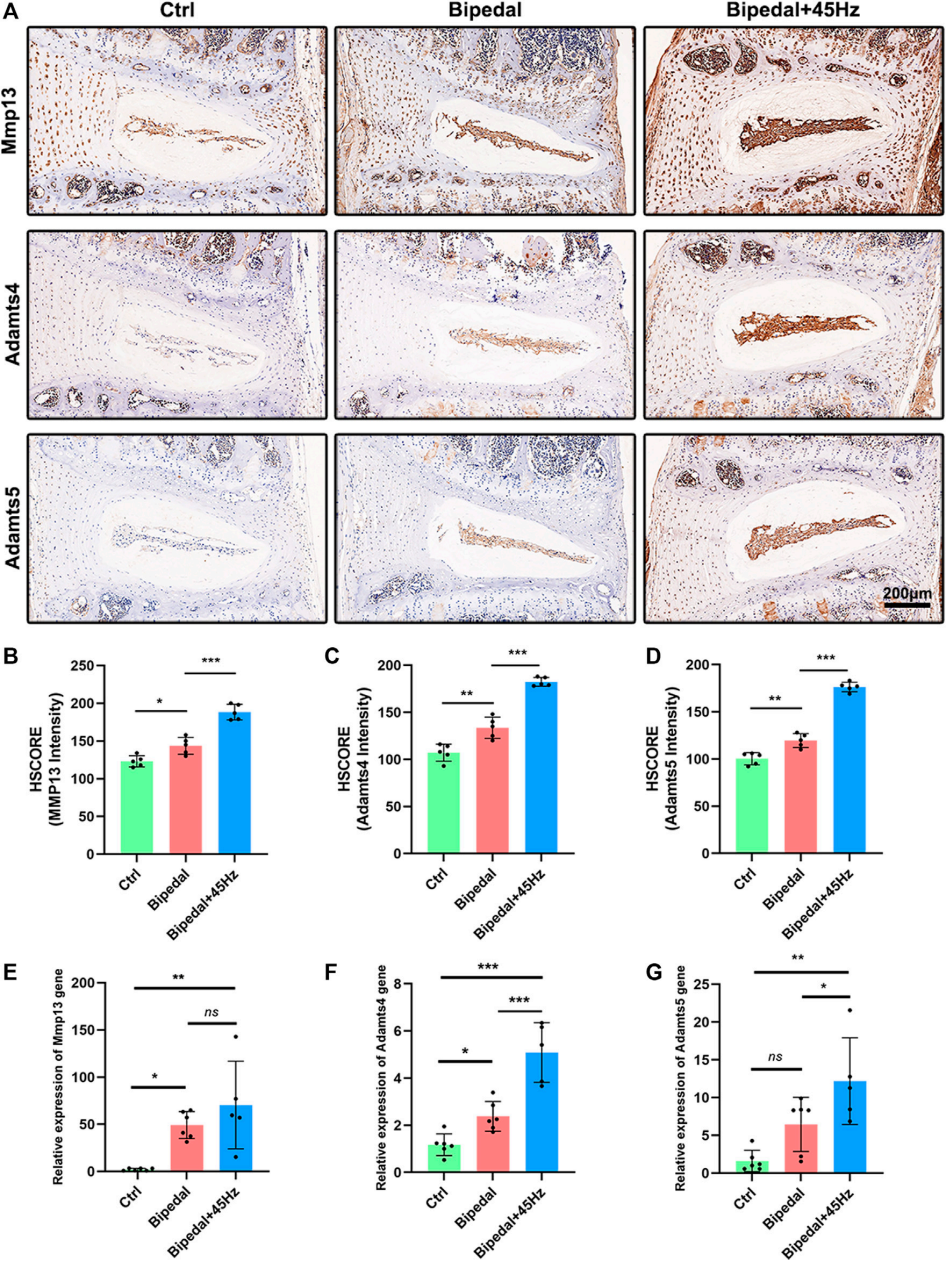
**(A)** The catabolic synthesis (Mmp13, Adamts 4, and 5) of IVD was marked using immunohistochemistry staining of these three groups. **(B–D)** The HSCORE results of these markers (*n* = 5 discs per group). **(E–G)** The analysis of IVD catabolic gene expression using qRT-PCR (*n* = 6 mice per group). *p < 0.05, **p < 0.01, ***p < 0.001, *ns* means no significant statistic difference.

To investigate the imbalance of synthesis and the level of catabolism of the extracellular matrix, we performed immunohistochemical staining and real-time PCR on the IVDs (Figures 3, 4). The IHC results of aggrecan, collagen I, and II showed no differences among these three groups. While the data of qPCR showed that WBV increased the gene expression of aggrecan and Col II, which were not in line with the IHC results. We next labeled the catabolism markers of IVD such as MMP13, Adamts4, and Adamts5. The results showed that 10 weeks of standing posture led to a significant high level of catabolism-associated protein compared to the control group. Furthermore, the long-term WBV treatment aggravated the level of catabolic proteins in the bipedal model. Similar to the IHC results for catabolism, gene expression was decreased in the bipedal group compared with control mice, and WBV conditions enhanced these genes expression (Figure 4).

**FIGURE 5:**
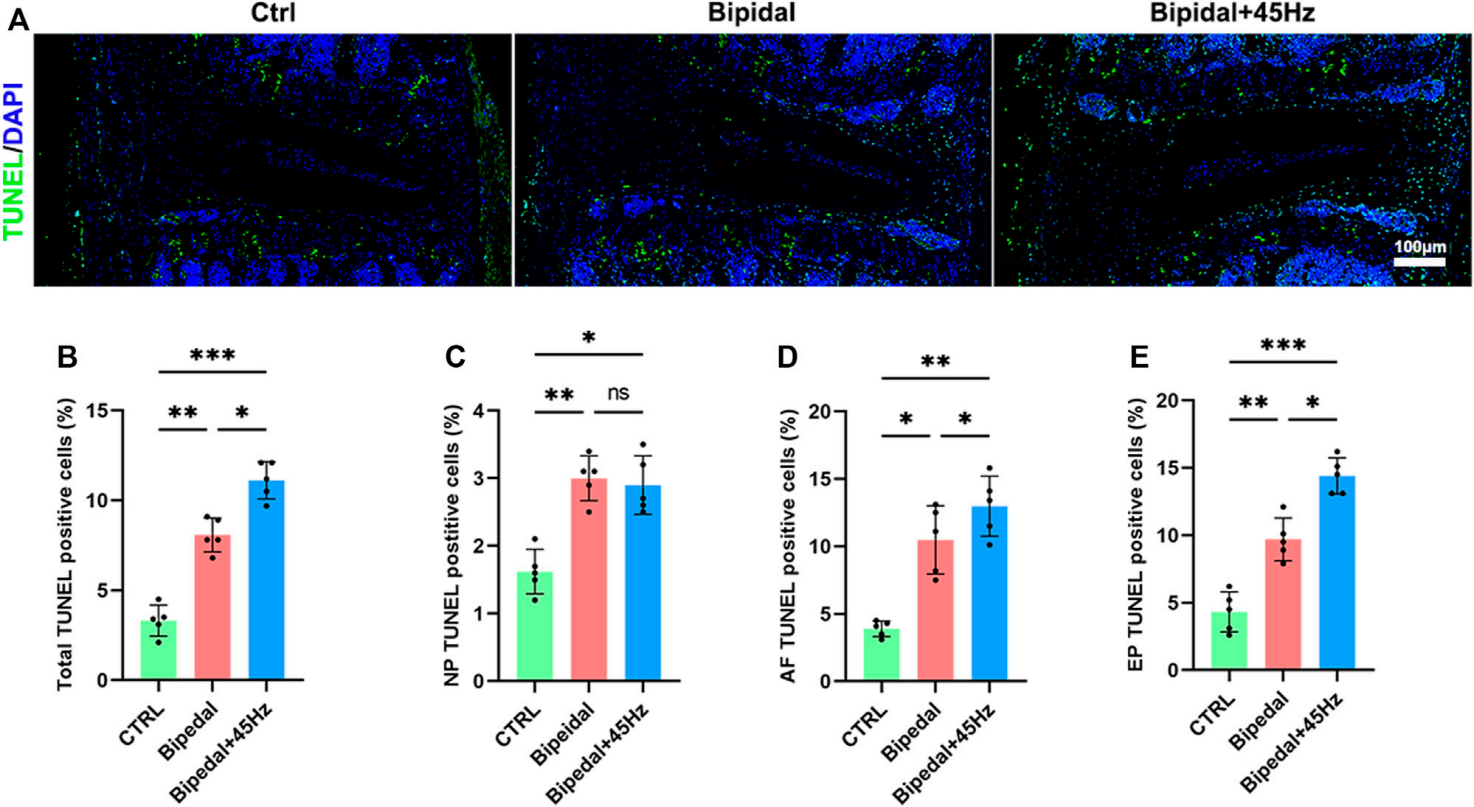
**(A)** TUNEL staining of IVDs. **(B)** Total TUNEL positive cells of total IVD tissues for each group. **(C)** The TUNEL positive cells of NP tissue for each group. **(D)** The TUNEL positive cells of AF tissue for each group. **(E)** The TUNEL-positive cells of EP tissue for each group. (*n* = 5 discs per group, *p < 0.05, **p < 0.01, ***p < 0.001, *ns* means no significant statistic difference, scale bar: 100 μm).

To determine whether bipedal and WBV altered cell viability in the IVD, TUNEL staining was taken on these IVDs (Figure 5A). Bipedal mice revealed a greater percentage of TUNEL-positive cells in the NP, AF, and EP compared to the control mice. Those bipedal mice exposed to WBV showed a greater percentage of TUNEL-positive cells in AF and EP compared to those bipedal mice without WBV treatment (Figures 5B–E).

**FIGURE 6:**
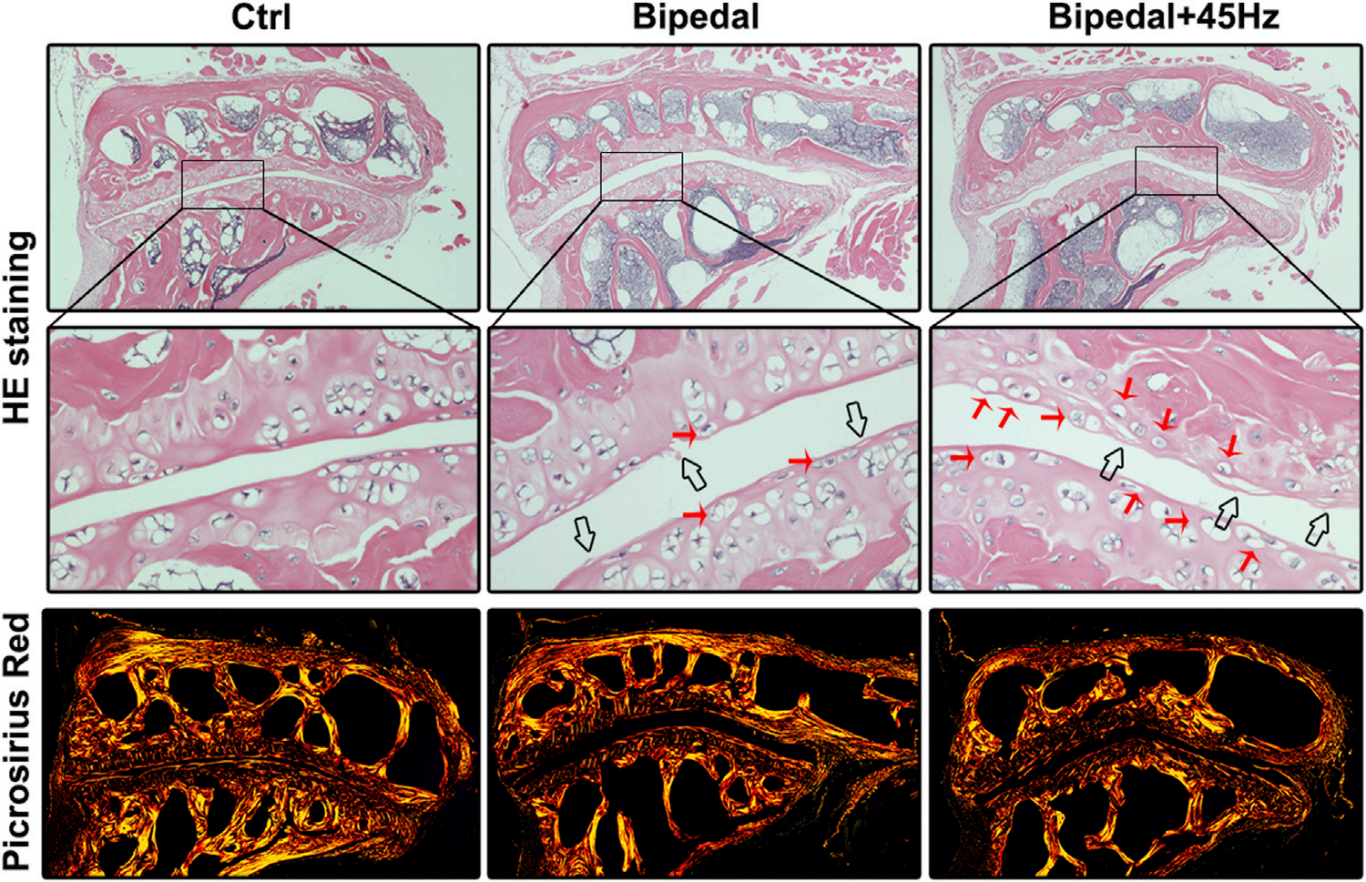
HE and Picrosirius Red staining showed that long-term standing posture could lead to the degeneration of FJ and WBV loading conditions could aggravate the changes of FJ degeneration, where FJ containing rougher surface of FJ and increased number of hypertrophic cells. Red arrows represent hypertrophic cells, and black arrows represent the rough surface of FJ.

**FIGURE 7:**
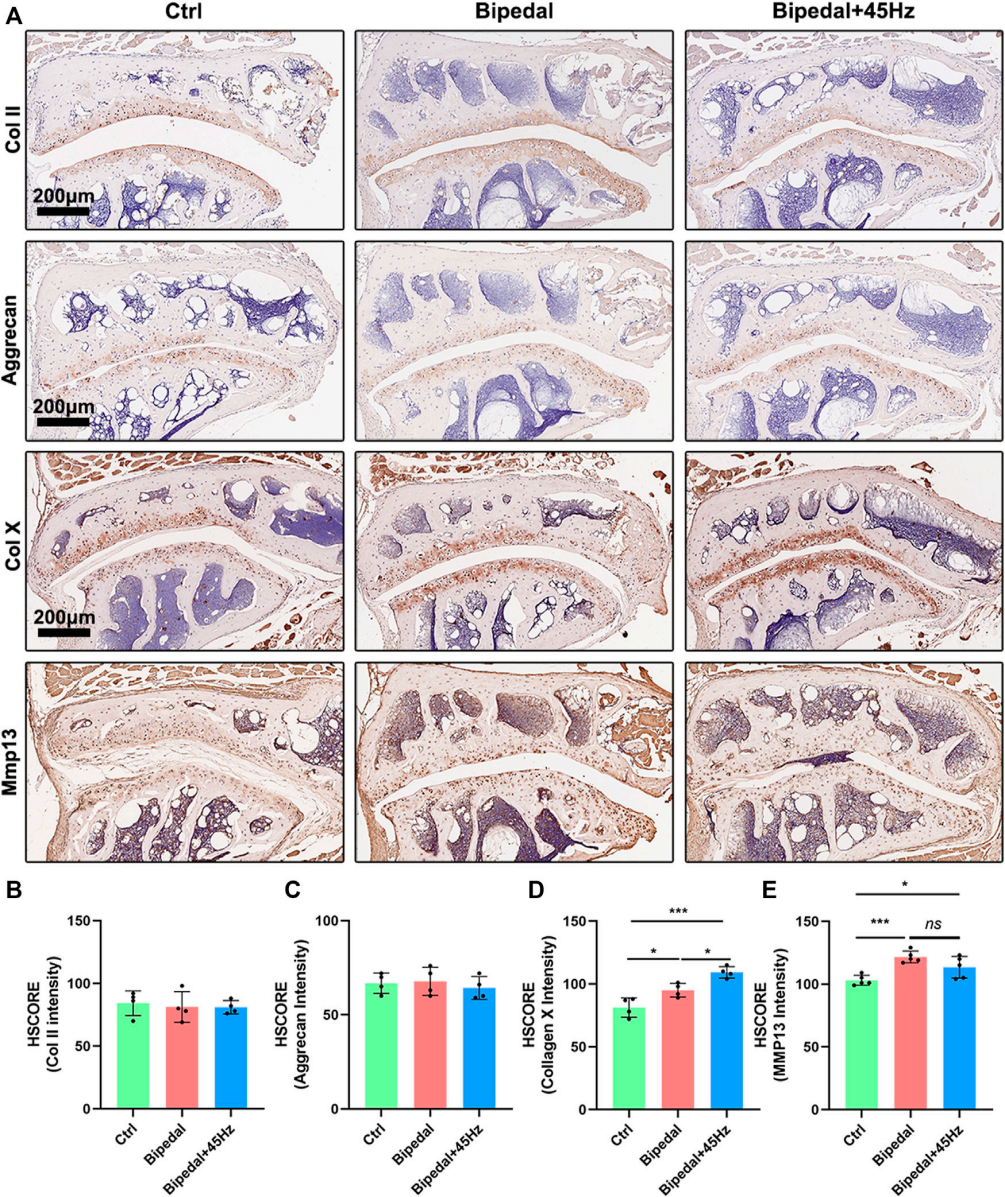
The anabolic and catabolic synthesis [**(A)**, Collagen II, Aggrecan, Collagen X, and Mmp13] of FJ were marked using immunohistochemistry staining of these three groups. **(B–E)** The HSCORE results of these markers (*n* = 4 per group). *p < 0.05, ***p < 0.001, *ns* means no significant statistical difference, scale bar: 200 μm.

**FIGURE 8:**
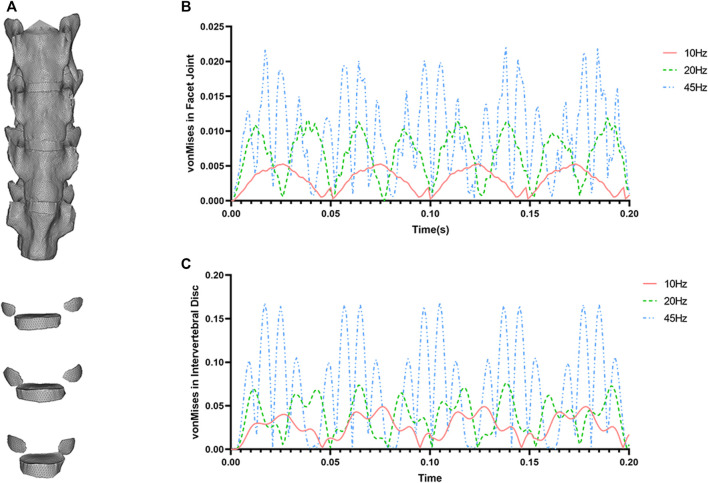
**(A)** The finite element model of mouse lumbar spine (L4-L6). **(B)** The von Mises compressive stress in FJ using different frequencies (10, 20, and 45 Hz) under vibration conditions. **(C)** The von Mises compressive stress in IVD using different frequencies (10, 20, and 45 Hz) under vibration conditions.

We next sought to investigate whether the deleterious changes detected within the IVD also occurred within the facet joint. Histologic examination revealed that FJs of bipedal mice were degenerated compared with control mice, and WBV treatment could accelerate these degeneration conditions. Specifically, the bipedal mice presented with several pathologic changes, such as a rough articular surface, formation of articular chondrocyte clusters, and increased hypertrophic chondrocytes on the articular surface (Figure 6). WBV aggravated these pathologic changes compared to the bipedal mice. The results of picrosirius red staining were consistent with those of the HE staining. Briefly, collagen organization was disrupted in the bipedal mice, and WBV led to a rougher surface of the FJs in the bipedal+45 Hz group. To explore the extracellular matrix metabolism of FJs exposed to standing postures with/without WBV, IHC staining of aggrecan, collagen II, X, and Mmp13 was performed (Figure 7). The level of synthesis markers of ECM (aggrecan and collagen II) did not present any differences among these groups (Figures 7B, C). However, the level of catabolism/hypertrophic markers (collagen X and Mmp13) were enhanced by the standing postures compared with control mice (Figures 8D, E). Furthermore, WBV also enhanced the level of collagen X in the present study (Figure 7D).

To investigate the biomechanical effects of WBV on the dynamic response of the IVD and FJ, a finite element model of the lumbar L4-L6 segment was successfully built. The upper body weight of 20 g (assuming gravity acceleration *g* = 10) was added on the superior surface of L4 vertebrae by means of a preload, then the 0.2N cyclic loads with different frequencies of 10, 20, and 45 Hz were imposed, as shown in Figure 8. After transient dynamic analysis, the data of compressive stress (von Mises stress) were acquired for further analyses. The maximum transient von mises stresses of IVD and FJ are smaller under 10 Hz WBV than those of 20 and 45 Hz. The von Mises of IVD and FJ at 45 Hz has the highest level among these three groups.

## Discussion

Whole body vibration has been used as an adjuvant treatment for a wide range of musculoskeletal diseases, including osteoporosis, low back pain, and osteoarthritis (Rubin et al., 2004; Herrero et al., 2011; Claerbout et al., 2012; Pang et al., 2013). However, conflicting results on the effects of WBV on regulating intervertebral disc matrix metabolism have recently been reported in mouse models (Wenger et al., 2010; McCann et al., 2013; McCann et al., 2015). Further, no studies have focused on the effects of WBV on facet joints in either animal models or humans till now. In addition, the posture of the mouse spine is quite different from human beings during daily life. As such, constructing an effective standing posture model could be helpful for studying the mechanical and biological metabolism of WBV on the IVD and FJ. Thus, the present work was designed to examine the effects of repeated WBV loading conditions on IVD and FJ in a novel bipedal mouse model.

In the present study, the water-escape habit of mice was used to build the bipedal model according to a previous study (Ao et al., 2019). This model promised a long-time standing posture during the building process of the model, and this posture successfully simulated standing and sitting postures of human beings for the spine of mice. Axial loading induced by bipedal posture simulated the process of IVD and FJ degeneration. Long-term standing mice presented qualitative pathological changes of IVD, such as a decreased number of NP cells, degeneration of the extracellular matrix, and a disorganized AF alignment. Further, by changing the directive distribution of axial stress using this bipedal model, the facet joint, an important posterior joint of the spine, showed a rough surface of joint and hypertrophic cells. Therefore, the current model could be used as an effective tool to investigate the metabolism of IVD and FJ and the effects of WBV on the spinal connect tissues. There is no consensus on specific WBV parameters (such as amplitude, frequency, or acceleration), as evidenced by apparently contradictory results in recent reports (Torcasio et al., 2008). The parameters of WBV (45 Hz, peak-to-peak amplitude of 74 μm, and 0.3 g peak acceleration) used in the present study were chosen according to previous studies (McCann et al., 2013; McCann et al., 2015) because these parameters brought beneficial effects of WBV on muscle and bone (Oxlund et al., 2003). Previous studies have shown beneficial results of the acute response of IVD tissues to WBV using *ex vivo* and *in vitro* mouse models. In brief, acute WBV led to a transient decrease in the expression of catabolism genes and an increased level of anabolic genes in IVD tissues (McCann et al., 2013). However, long-term repeated exposure to WBV resulted in degeneration of IVD and knee joint using CD-1 mouse, but repeated exposure to WBV in C57BL/6 mouse did not present damage performance for IVD and knee joint (McCann et al., 2015). One possible explanation for the different results is that the resonant frequency may play a role in response to WBV, because the resonant frequency is a function of animal/tissue mass (Rabey et al., 2015; Patterson et al., 2022). Another explanation is that the posture may play an important role in the response to WBV in different type of mouse.

Interestingly, the current study demonstrated that long-term standing posture leads to degeneration of the IVD in mice, with a decreased height of IVD and a number of NP cells, and disordered arrangement of annulus fibrosus, which were in line with previous results (Ao et al., 2019). The bipedal posture changed the stress distribution on the mouse spine, which was similar to human beings. Then, the axial mechano-stimulations directly increased the stress on the IVD and FJ. Meanwhile, the elevated pressure of IVD was converted into tension applied to the AF regions. Furthermore, repeated WBV may accelerate the procedures of IVDD and resulted in decreased DHI and number of NP cells, and disorder of AF lamellae. One of the explanations for this change was that AF exposure to vibration may directly result in injury of the AF matrix (Gregory and Callaghan, 2012). Our results were consistent with those characterized effects of dynamic compressive stress on the IVD. Although studies have reported that the structural changes predominantly occurred in the NP region, dynamic loading situations were shown to increase the anabolism and apoptosis of AF *in vivo* (Gregory and Callaghan, 2012). These studies reported different effects of dynamic mechano-loading on different compartments of IVD, which may attribute to different levels of stress and frequency of the applied loading conditions. However, these studies did not consider the influence of postures on IVD. Thus, further studies are needed to determine whether the response of IVD to WBV may also be influenced by different parameters of vibration using the bipedal model.

In the present study, the IHC results revealed no obvious changes of anabolism (collagen I, II, and aggrecan), and increased level of catabolism (such as Mmp13, Adamts4, and Adamts5) in the IVD of bipedal mice. Regarding anabolism, Ao et al. (2019) reported a decreased level of collagen II in the IVD of bipedal mice compared to control mice. The anabolic markers (aggrecan, Col I and II) are the larger protein with mechanical function in the IVD (Liu et al., 2017). While our data showed no difference in the effect on the anabolic protein level in the bipedal model. This may be attributed to the age of the mice we used which were younger than those of previous studies (Ao et al., 2019), where younger mice have a stronger ability to restore the normal level of anabolism. Dynamic stress on the IVD tissue has been reported beneficial effects on the anabolic genes in several *in vitro* and *in vivo* studies (Kasra et al., 2003; Liang et al., 2017), and the application of WBV in the hospital as a therapy method for patients associated with musculoskeletal diseases, acute whole-body vibration has been reported to show beneficial effects on the anabolism of IVD in laboratory research (McCann et al., 2013). Whereas, the bad effects of long-term exposure to WBV were also shown using the same parameters (McCann et al., 2015). Here, in the present study, WBV did not promote the degeneration of anabolic protein level (Col I, Col II, and aggrecan) in the bipedal mice, but could promote the genes (Col I, Col II, and aggrecan) expression level. Given the controversial results about the anabolism of IVD responding to mechanical stimulates, it is possible that the frequency, amplitude, time, types, and directions of stress provide different effects on the anabolism of IVD. Regarding catabolism, changes in the histologic presentation of the IVD induced by standing postures were accompanied by increased catabolism levels, as evidenced by the accumulation of catabolic markers (Mmp13, Adamts4/5). Further, axial WBV conditions promoted the catabolism level of IVD in the bipedal model. Mmp13, Adamts4, and Adamts5 were the major catabolic marker for IVD (McCann et al., 2013), and the Mmps expression level is associated with degenerative lumbar stenosis (Omair et al., 2013). The bipedal mouse model demonstrated an obviously high catabolic level in the intervertebral disc. Our data were consistent with these results. Short-term WBV loading conditions have been reported to show no effects on the Adamts4/5 gene expression of IVD(25), while long-term exposure to WBV could lead to IVDD by promoting the level of catabolism (McCann et al., 2015). Taken together, these findings suggest that catabolic markers are regulated by axial WBV conditions in IVD dependent on time, direction, frequency, amplitude, and parameters of whole-body vibration stimulation.

In the facet joint, we detected articular cartilage damage (rough surface and increased number of hypertrophic cells) in the facet joint after spending a long time in a standing posture. The degeneration and arthritis of the facet joint were considered clinically important reasons for LBP (Beresford et al., 2010). The loading stress on the lumbar spine of quadrupeds is different from that of human beings. Thus, it is not appropriate to investigate the biomechanical microenvironment of the lumbar spinal segments in mice. Therefore, it is necessary to establish a novel animal model to simulate the daily posture of humans to establish the biomechanical loading conditions, because it is an essential step in the effort to explore the pathological changes of FJ degeneration associated with mechanical stress. Here, the current study uses a novel bipedal mouse model, which could simulate human standing postures for a long time (Ao et al., 2019; Li et al., 2020). Using this novel model provides natural positions resembling human beings, and gives researchers a chance to simulate the mechanical loadings in animal studies. In the present study, upright posture directly leads to a rough articular surface and an increased number of hypertrophic chondrocytes, and after the long-term WBV loading on the FJ in the standing mice, the aberrant stress loading acted on the facet joint accelerated degeneration of FJ and increased level of hypertrophic markers. Collagen X and Mmp13 were important biomarkers for degeneration and hypertrophy of cartilage. These increases in Col X and Mmp13 levels may represent a cellular response to WBV and finally lead to a higher level of catabolism compared to bipedal mice and control mice.

As with the lumbar intervertebral discs, there are few studies focused on evaluating the effect of WBV on the lumbar facet joint. Recent reports described several methods to establish the FJ arthritis model in large animals and mice (Wang et al., 2018; Ni et al., 2019). However, they were quite different from the natural pathogenesis of FJ degeneration induced by biomechanical microenvironment changes. The bipedal model used in the present study could simulate human postures and provide a new experience in assessing the effects of WBV on lumbar segments. Our findings demonstrated that long-term exposure to axial WBV loading conditions could deteriorate the degeneration of the lumbar facet joint.

The effects of WBV were confused and contradictory according to previous reports. However, long-time WBV has been found to cause health risks for lumbar motion segment L3-L5 (Hulshof and van Zanten, 1987; Pankoke et al., 2001). Studies have shown that dynamic loading conditions were more harmful and could promote stress, displacement, damaging forces in lumbar segments (Kasra et al., 1992; Keller et al., 2002; Guo et al., 2005). Considering the degeneration changes of IVD and FJ caused by long-term WBV in the present study, we further built a 3-dimensional finite element model of mouse lumbar segment L3-L5 to investigate the dynamic effects of WBV on the lumbar spine, which, we hope, could partially explain the biomechanical mechanism of histological changes caused by whole body vibration in the present study. From the results of the present model analyses, it can be found that the amplitudes of dynamic compressive stress are increasingly large in line with the frequencies. Thus, different stimulating WBV loading conditions might affect the catabolism of the IVD and lead to degeneration under long-term exposure. Taken together, our study demonstrated that long-term WBV on the lumbar segments (including IVD and FJ) suffered from higher stresses at 45 Hz compared to the frequency of 10Hz, which might cause disorders and degeneration of the IVD and FJ.

There are several limitations to our study. The posture of this model is inconsistent with the natural loading direction of the mouse spine, which might contribute to the aberrant changes in the spinal tissues of the mouse. Furthermore, this model allows increased loading without the external constraint of range of motion, and the compensatory mechanisms, such as spinal muscle, may present different extended stress models on the IVDD and FJ induced by WBV conditions. A novel model, allowing strictly controled parameters and more closely representing the human sequence, should be developed and used in future studies to investigate the effects of WBV on IVD and FJ.

In conclusion, the results of the present study have shown that long-term exposure to WBV caused degeneration of both the IVD and FJ of the lumbar segments in a bipedal mouse model. We first investigated the effects of whole-body vibration at the axial orientation using a non-invasive bipedal mouse model of intervertebral disc degeneration. This model provided us with a new tool and method to observe the responses of IVD and FJ to vibration conditions in a bipedal posture. Our findings raise concerns about the clinical use of the WBV platforms for treating various diseases, as well as exposure to vibration conditions in daily life, and axial WBV could accelerate the degeneration of the intervertebral disc and facet joint. Furthermore, studies are needed to investigate the effects of different frequencies and amplitudes of WBV on the lumbar segments.

## Data Availability

The original contributions presented in the study are included in the article/Supplementary Material, further inquiries can be directed to the corresponding authors.

## Supplementary material

SUPPLEMENTARY FIGURE S1
**(A)** Micro-CT results show that the intervertebral height of the bipedal group and bipedal+45Hz group were decreased compared to those of the control group. **(B)** The diagram of the measurement of disc height index (DHI%), where A-C represent the height of the anterior 25%, 50%, and 75% of the intervertebral disc and D-I represents the height of 25%, 50%, and 75% of the adjacent vertebrae. **(C)** The DHI% of the mice among these three groups was shown. (*n* = 5 discs per group, **p < 0.01, ***p < 0.001 compared with the control group).
