# Supplementary figures and images for: Long-term whole-body vibration induces degeneration of intervertebral disc and facet joint in a bipedal mouse model
Source: Front Bioeng Biotechnol. 2023 Mar 17;11:1069568. doi: 10.3389/fbioe.2023.1069568 (PMC10063969; doi:10.3389/fbioe.2023.1069568)

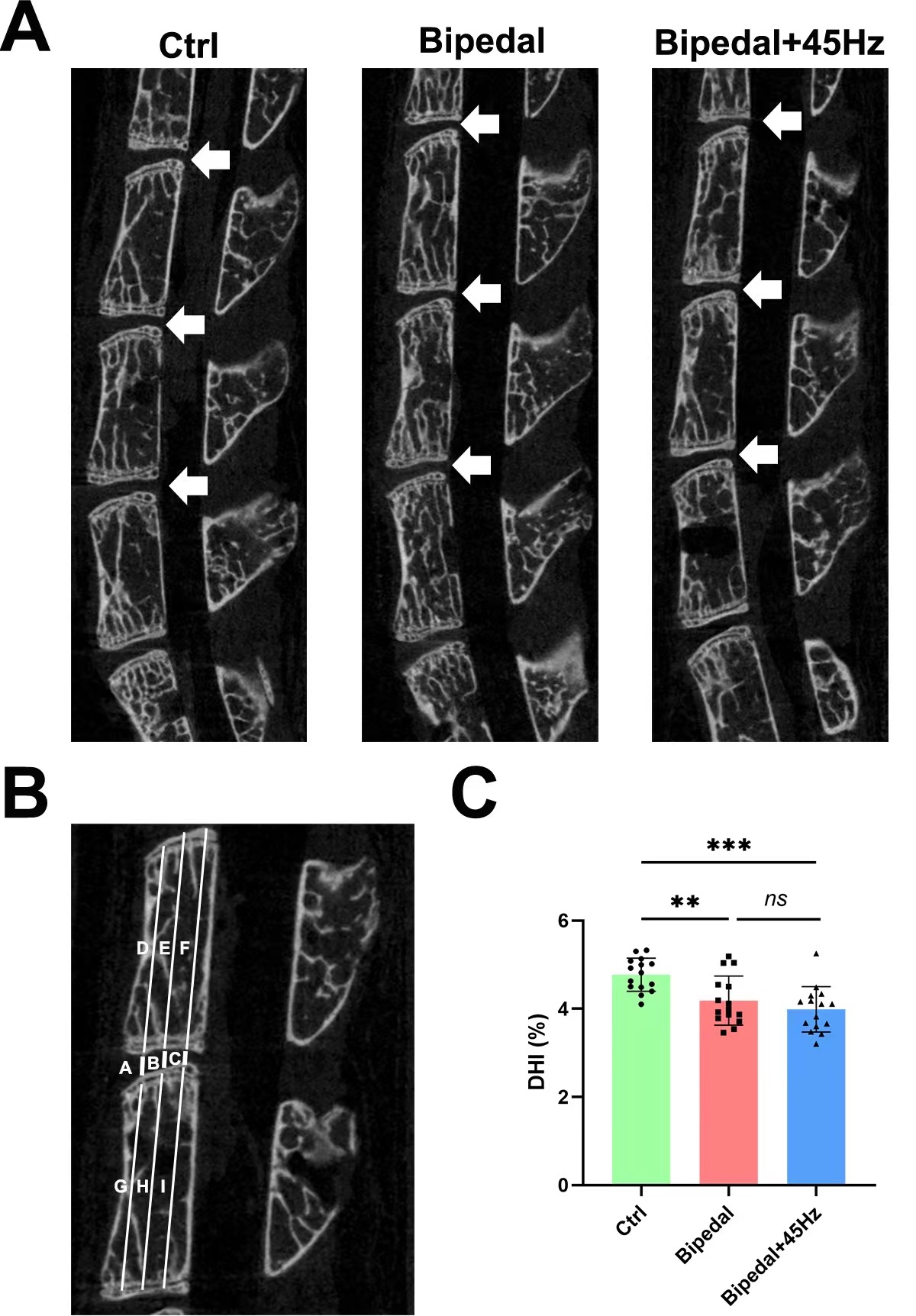

Supplement: Supplementary file 1 [file Image1.jpg]
